# Autophagy induced by a sulphamoylated estrone analogue contributes to its cytotoxic effect on breast cancer cells

**DOI:** 10.1186/s12935-016-0367-5

**Published:** 2016-12-08

**Authors:** Marcel Verwey, Elsie M. Nolte, Anna M. Joubert, Anne E. Theron

**Affiliations:** Department of Physiology, Faculty of Health Sciences, University of Pretoria, Private Bag X323, Arcadia, Pretoria, 0007 Gauteng South Africa

**Keywords:** Breast cancer, Autophagy, Apoptosis, Cell survival, ESE-15-ol, 3-Methyladenine

## Abstract

**Background:**

Autophagy can either be protective and confer survival to stressed cells, or it can contribute to cell death. The antimitotic drug 2-ethyl-3-*O*-sulpamoyl-estra-1,3,5(10),15-tetraen-17-ol (ESE-15-ol) is an in silico-designed 17-β-estradiol analogue that induces both autophagy and apoptosis in cancer cells. The aim of the study was to determine the role of autophagy in ESE-15-ol-exposed human adenocarcinoma breast cancer cells; knowledge that will contribute to future clinical applications of this novel antimitotic compound. By inhibiting autophagy and determining the cytotoxic effects of ESE-15-ol-exposure, deductions could be made as to whether the process may confer resistance to the drug, or alternatively, contribute to the cell death process.

**Methods and results:**

Spectophometrical analysis via crystal violet staining was used to perform cytotoxicity studies. Morphology studies were done using microscopic techniques namely polarization-optical transmitted light differential interference light microscopy, fluorescent microscopy using monodansylcadaverine staining and transmission electron microscopy. Flow cytometry was used to quantify the autophagy inhibition and assess cell viability. Results obtained indicated that 3-methyladenine inhibited autophagy and increased cell survival in both MCF-7 and MDA-MB-231 cell lines.

**Conclusion:**

This in vitro study inferred that autophagy inhibition with 3-methyladenine does not confer increased effectiveness of ESE-15-ol in inducing cell death. Thus it may be concluded that the autophagic process induced by ESE-15-ol exposure in MCF-7 and MDA-MB-231 cells plays a more significant role in cell death than conferring survival.

## Background

**Fig. 1:**
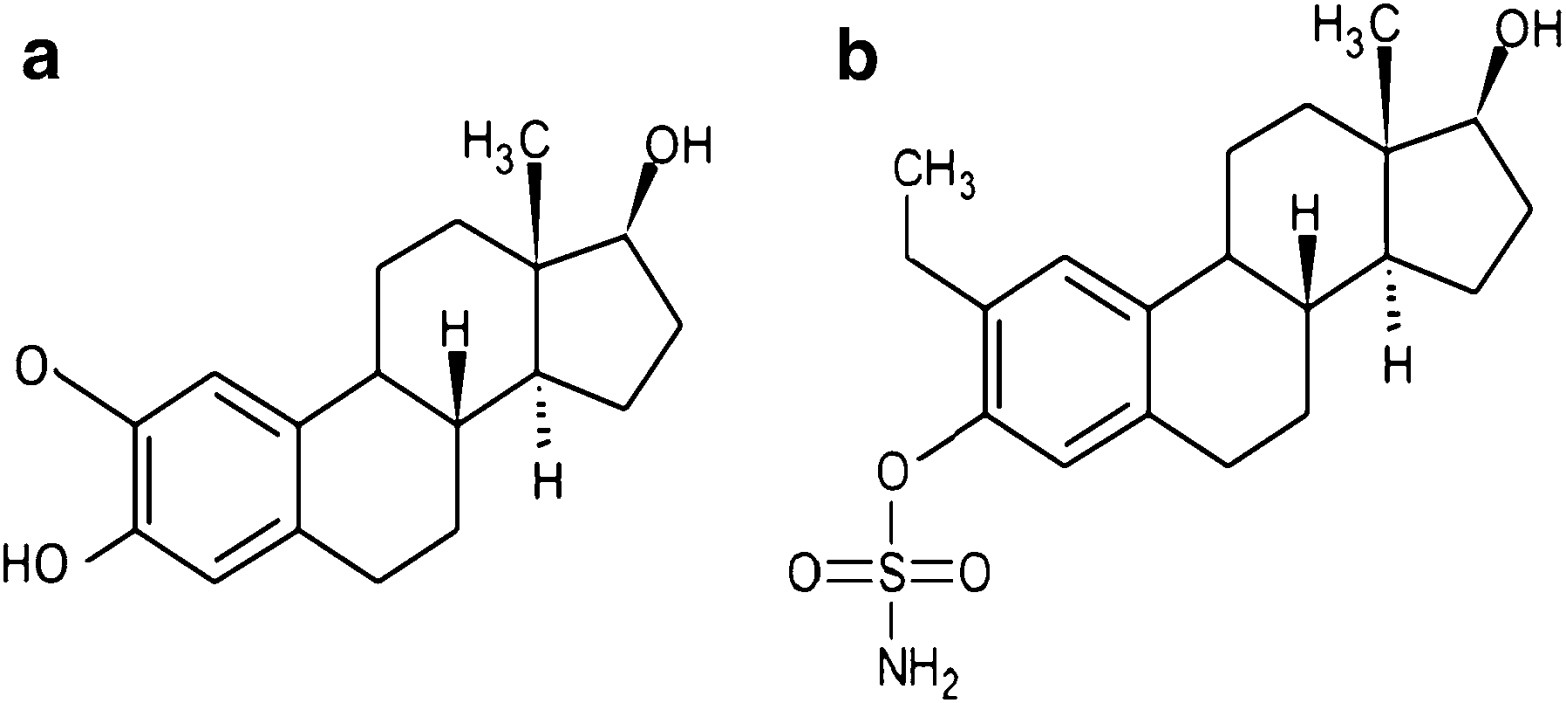
Structure of 2-methoxyestradiol and ESE-15-ol. **a** 2-Methoxyestradiol is the parent compound of ESE-15-ol; **b** ESE-15-ol is a novel sulphamoylated estradiol analogue

2-Methoxyestradiol (2ME), a microtubule depolymerising agent, is both an anti-cancer and anti-angiogenic drug that has shown promise in cancer research (Fig. 1a) [1–3]. Although it is formed through the sequential endogenous metabolism of 17-β-estradiol, the compound exerts its cytotoxic effect independently of the cellular estrogen receptors and has no significant systemic hormonal effects [1–4]. 2ME is able to inhibit cancer cell proliferation, whereas estradiol promotes proliferation of cancer cells. 2ME inhibits hypoxia-inducible factor-1α (HIF-1α) which, in return causes inhibition of angiogenesis, as well as disruption of microtubules [2, 5]. 2ME causes both the intrinsic- and extrinsic apoptotic pathways to be up-regulated by decreasing B-cell lymphoma 2 (Bcl-2) which has anti-apoptotic properties, or by increasing the death receptor 5 (DR5) [2, 5]. Actively proliferating cells, such as cancer cells, are the main target for this drug-induced apoptosis while preferential sparing of normal, quiescent endothelial cells is observed [3, 6]. 2ME not only causes a G_1_ cell cycle arrest, but also a G_2_/M arrest [3, 7]. However, the molecular mechanism induced by 2ME differs between cell lines [5]. 2ME has undergone clinical trials for solid tumors, but shows limited bioavailability and rapid degradation in vivo [1–3, 8].

2-Ethyl-3-*O*-sulpamoyl-estra-1,3,5(10),15-tetraen-17-ol (ESE-15-ol), a derivative of 2-ethyl-3-*O*-sulpamoyl-estra-1,3,5(10),15-tetraen-17-one (ESE-15-one) is a novel sulphamoylated estradiol analogue (Fig. 1b) [9]. ESE-15-ol is an anti-mitotic compound that binds to the colchicine binding site on microtubules, and is equally effective in both hormone-receptor positive- and negative cancer types [9–11]. ESE-15-ol induces both autophagy and apoptosis in breast cancer cells [9]. This potential anticancer drug was in silico-designed to increase the parent compound’s bioavailability via carbonic anhydrase II (CAII) binding by the addition of a sulphamate group at the C′3 position [9]. The latter enzymatic binding occurs in erythrocytes and results in a slower release of drugs into the circulation, thereby bypassing the hepatic first-pass metabolism [9, 12, 13]. Tumors have acidic micro-environments in which CAIX convert carbon dioxide (CO_2_) to carbonic acid [14]. This acidic environment enhances metastatic spread due to proteinase release [9, 15]. Therefore the molecule was designed to also have an increased binding affinity to CAIX over CAII. By binding to CAIX, ESE-15-ol should selectively locate to solid tumours and potentionally improve chemotherapy by reducing the acidic surrounding, thereby decreasing metastasis [9].

Apoptosis is an energy-dependant mode of cell death and is also known as programmed cell death type I [16–18]. If apoptosis is down-regulated it permits tumor growth and multi-drug resistance [18]. Type II-programmed cell death is a degradative process known as autophagy which is associated with the formation of autophagic vesicles [19, 20]. Literature points at a possible dual role associated with autophagy since it can contribute to either cell survival or cell death, depending on a myriad of different conditions [19, 20]. It may promote survival by facilitating an adaptive response to cellular stress through providing an alternative source of energy during starvation. Additionally, autophagy can increase cell survival by protecting them from apoptosis through the down-regulation of pro-apoptotic proteins [19]. However, prolonged autophagy can lead to cell death due to the high protein turnover rate [21]. Autophagy and apoptosis are interconnected and share common stimuli for the execution of both pathways. Apoptosis and autophagy can thus either have synergistic or antagonistic effects [22].

3MA, a nucleotide derivative, is an inhibitor of autophagy [23]. 3MA blocks autophagy through inhibiting class I and class III phosphatidylinositide-3-kinases (PI3K) [23, 24]. Class III PI3K is a lipid protein that phosphorylates the 3rd position on the inositol ring in phosphatidylinositol to form phosphatidylinositol-3-phosphate (PI3P), which is essential for the initial steps in autophagy [23]. This leads to the activation of protein kinase B (Akt) which then phosphorylates the mechanistic target of rapamycin (mTor) [20, 23]. 3MA can suppress cell invasion and migration of fibrosarcoma cells (HT1080) by inhibiting class III PI3K [25].

Studies by Xie et al. [27] and Li et al. [26] have shown that autophagy inhibition by 3-methyladenine (3MA) increased apoptotic cell death in human colon cancer cell lines and human hepatoma cells (HepG2). By inhibitiong the protective mechanism of autophagy, chemoresistance was overcome. However, Bonet-Ponce et al. [28] demonstrated that inhibition of autophagy by 3MA inhanced cell survival through reducing the oxidative stress-induced cell death. This indicates that the mechanisms of autophagy are cell and drug dependant.

In this study autophagy inhibition with 3MA during ESE-15-ol exposure was conducted to allow insight into whether autophagy will confer resistance to drug-exposed cells, or whether it will contribute to programmed cell death.

## Methods

### Reagents

Dulbecco’s minimum essential medium Eagle (DMEM), trypsin–EDTA, 3-methyladenine (3MA) and all other reagents not specifically mentioned were of analytical grade and purchased from Sigma-Aldrich (St. Louis, USA). Streptomycin, fungizone and penicillin were manufactured by Thermo Fisher Scientific (Massachusette (MA), USA). Crystal violet, gluteraldehyde and triton X-100 were purchased from Merck (Darmstadt, Germany). Anti-LC3B/MAP1LC3B-antibody was supplied by Novus Biological [Littleton, Colorado (CO), USA].

### Chemical compounds and appropriate controls

The novel estradiol analogue, ESE-15-ol, was synthesized by Ithemba Pharmaceuticals (PTY) Ltd. (Modderfontein, Gauteng, South Africa) as it is not commercially available. A working 1 mM stock solution of ESE-15-ol in dimethyl sulfoxide (DMSO) was prepared at the Department of Physiology, University of Pretoria and stored at −20 °C. DMSO was used as a vehicle control, never exceeding a 0.05% concentration in the final dilution. Actinomycin D (Sigma-Aldrich, St Louis, USA) was used as a positive control for apoptosis at a final concentration of 0.1 μg/ml. A final concentration of 20 μM tamoxifen (Sigma-Aldrich, St Louis, USA) was used as a positive control for autophagy. Tamoxifen (20 μM) combined with 5 mM 3MA was used as a positive control for autophagy inhibition. All chemicals were of analytical grade and purchased from Sigma-Aldrich (St Louis, USA), unless otherwise stated.

### Cell lines and general cell culture protocols

For this in vitro study, the human adenocarcinoma breast cancer MCF-7 cell line and the metastatic human adenocarcinoma breast cancer MDA-MB-231 cell line were used (Cellonex, Johannesburg, South Africa). Penicillin G (100 U/ml), fungizone (250 μg/l), streptomycin (100 μg/l) and 10% heat activated fetal calf serum (FCS) (Gibco^®^ Invitrogen, California, USA) were added to DMEM. For the 96 well plates, 5000 cells were seeded in 200 μl growth medium in each individual well. For the 6 well plates, 375,000 cells were seeded in 3 ml growth medium in each individual well. For the 25 cm^2^ culture flasks, 1 × 10^6^ cells/5 ml growth medium were seeded. Cells were incubated in a Forma Scientific water-jacketed incubator (Ohio, USA) in a humidified atmosphere at 5% CO_2_ (37 °C) for 24 h before exposure to ESE-15-ol, with or without 3MA. A final concentration of 5 mM 3MA was used to inhibit autophagy during exposure with ESE-15-ol or tamoxifen. Cells were pre-exposed to 3MA for an hour prior to addition of ESE-15-ol or tamoxifen.

### Spectrophotometry: crystal violet

Crystal violet is a triarylmethane dye (purple) that stains the deoxyribonucleic acid (DNA) within cells in a monolayer culture. Cytotoxicity of a drug may be determined by measuring the absorbance of the crystal violet stained cells via spectrophotometry. The half maximal growth inhibitory concentration (GI_50_) may thus be determined [29, 30]. After a 24 h-exposure to an ESE-15-ol dose concentration series, 100 μl 1% gluteraldehyde was added to the cells and samples were incubated for 15 min at room temperature. Gluteraldehyde was discarded and 100 μl 0.1% crystal violet was added for 30 min for staining to take place. Crystal violet was discarded and the plate was washed under running tap water (±10 min). Plates were left to dry for 24 h. Triton X-100 (0.2%) (200 μl) was added to the wells and samples were incubated for 40 min at room temperature for solubilisation. After incubation, 100 μl of the solution was pipetted to a clean 96 well plate. Absorbance was read using the ELx800 Universal Microplate Reader (Bio-tek Instruments Inc. Vermont, USA) at 570 nm.

### Polarization-optical transmitted light differential interference microscopy

Viable cells were seeded at a density of 375,000 cells/3 ml growth medium in 6 well plates. After attachment, cells were exposed to ESE-15-ol in the presence or absence of 3MA for 24 h (37 °C) along with appropriate controls. PlasDIC images were viewed at a 40× magnification with a Zeiss Axiovert-40 microscope (Göttingen, Germany) and captured with the Zeiss Axiovert MRm monochrome camera (Göttingen, Germany).

### Fluoresence microscopy

Cells were exposed to ESE-15-ol in the presence or absence of 3MA for 24 h (37°) along with the appropriate controls. Cells were stained with 0.05 mM monodansylcadaverine (MDC) in PBS for 10 min (37 °C) and protected against light after which cells were washed four times with PBS. Using the Zeiss inverted Axiovert CFL 40 microscope and the Zeiss Axiovert MRm monochrome camera (Göttingen, Germany) fluorescent images were visualized with a UV filter with excitation of 380 nm and emission of 480 nm at a 40× magnification.

### Transmission electron microscopy

After 24 h of exposure to ESE-15-ol with or without 3MA alongside all the appropriate controls, cells were trypsinized and fixed in 2.5% gluteraldehyde in 0.075 M phosphate buffer for 1 h (room temperature). Cells were washed three times for 10 min with 0.075 M phosphate buffer followed by fixation in 0.5% aqueous osmium tetroxide for 1 h (room temperature). Cells were washed 3 times in distilled water. Increasing ethanol concentrations (10, 30, 50, 70 and 100%) were used to dehydrate the fixed cells for 10 min in each concentration. Quetol (30%) in ethanol was used to infiltrate the cells (1 h) followed by 60% quetol (1 h) and lastly in 100% quetol (4 h). Specimen were embedded and polymerized (60 °C, 39 h). Ultra thin sections were prepared using a microtome and each section was mounted on a copper grid. Samples were contrasted in 4% aqueous uranyl acetate (10 min) and then in Reynolds lead citrate (2 min). To view the TEM images the JEM-2100F field emission transmission electron microscope (JEOL, Tokyo, Japan) was used. All Chemicals provided by the Electron Microscopy unit, University of Pretoria.

### Cell cycle progression

Propidium iodide (PI) is a fluorescent dye used to quantify DNA content at 488 nm excitation [31]. Flow cytometry employing PI was used to distinguish cells in the different cell cycle phases. After exposure, cells were trypsinized and washed in 1 ml ice-cold PBS and resuspended in 200 μl ice-cold PBS. Cells were fixed in 4 ml ice-cold 70% ethanol, added drop wise while vortexing and incubated overnight at 4 °C. Cells were centrifuged (5 min at 100×*g*) and the pellet resuspended in PBS containing 0.01% triton X-100, PI (40 μg/ml) and RNase A (100 μg/ml) and incubated for 40 min at 37 °C. PI fluorescence was measured using flow channel 3 (FL3) on the FC500 system flow cytometer (Beckman Coulter, California, USA) excited at 488 nm. A minimum of 10,000 cells were analized using three biological repeats. Cell cycle distributions were analized with Cyflogic flow cytometry analysis software (Beckman Coulter, California, USA) using the DNA content per cell which illustrates the sub-G_1_, G_1_, S, G_2_/M fractions.

### Apoptosis detection: annexin-V FITC

Viable cells contain phosphatidylserine (PS), located on the inside of cell membrane. When apoptosis occurs, PS flip will occur. PS moves to the outside surface of cells to which annexin-V binds [32]. Washed cells were centrifuged and the supernatant discarded. The pellet was resuspended in annexin-V FITC binding buffer (0.25–10^7^ cells/ml). Cell suspension (100 μl) was pipetted into a 5 ml test tube. Annexin-V FITC (5 μl) and 10 μl of PI was added and samples were incubated at room temperature (15 min) in the dark. After incubation 400 μl annexin-V binding buffer was added to each test tube. PI (FL3) and annexin-V (FL1) fluorescence was analyzed with the FC500 system flow cytometer (Beckman Coulter, California, USA) equipped with an air-cooled argon laser excited at 488 nm. Three biological repeats with a minimum of 10,000 cells were analized. Data was analyzed with Cyflogic flow cytometry analysis software (Beckman Coulter, California, USA) with PI plotted on the x-axis and annexin V-FITC on the y-axis.

### Autophagy detection: microtubule-associated protein 1A/1B-light chain 3

To detect autophagy, flow cytometry was used employing microtubule-associated protein 1A/1B-light chain 3 (LC3) using a conjugated rabbit polyclonal anti-LC3B antibody which can be detected in FL1 (excitation = 488 nm; emission = 525 nm) [33]. After 24 h of exposure to ESE-15-ol with or without 3MA, washed trypsinized cells were resuspended in ice-cold PBS containing 0.01% formaldehyde and samples were incubated for 10 min at 4 °C. Cells were centrifuged and resuspended in 200 μl PBS to which 1 ml ice-cold 100% methanol was added and incubated for 15 min at 4 °C. Cells were washed twice with PBS. Cell pellet was resuspended in 200 μl PBS containing 0.05% triton X-100, 1% BSA, 40 μg/ml PI and anti-LC3B/MAP1LC3B-antibody (1:200) (Novus Biological, Littleton, CO, USA) and incubated for 2 h at 4 °C. Cells were washed twice with 1 ml PBS containing 0.05% triton X-100 and 1% BSA. LC3 fluorescence was measured in flow channel 1 (FL1) on the FC500 system flow cytometer (Beckman Coulter, California, USA). Three biological repeats with a minimum of 10,000 cells were used and data was analyzed with Cyflogic flow cytometry analysis software (Beckman Coulter, California, USA). Any cell debris and cell clumps were removed from analysis.

### Western blot analysis: LC3II

After 24 h exposure, DMEM was aspirated and cells were washed with ice-cold PBS. 200 μl RIPA cell lysis buffer (150 mM NaCl, 0.1% SDS, 10 mM Tris–HCl 0.5% sodium deoxcylate, 1 mM EGTA and 1 mM EDTA in ddH_2_0, adjusted to a pH of 7.4) was added to cells and incubated for 5 min on ice. Lysed cells were scraped and centrifuged for 30 min at 4 °C (1000×*g*). Protein concentration was determined by use of the Pierce^®^ BCA protein assay kit (Thermo Fisher Scientific Inc., Rockford, Illinois, USA). In a 96 well plate, 20 μl of the cytosolic extract was added to 100 μl of the protein assay working solution. The absorbance was read at 570 nm by use of ELx800 Universal Microplate Reader (Bio-tek Instruments Inc. Vermont, USA). The protein concentration was calculated by using a standard curve. After protein quantification 25 μg protein of each sample, containing 5% b-mercaptoethanol and NuPAGE LDS buffer (1:4) (Sigma-Aldrich, St. Louis, USA) was denaturated at 96 °C (10 min). Samples (20 μl) were loaded into NuPAGE 4–12% Bis–Tris gel wells alongside a relevant protein band size ladder. Samples were run in 1× MOPS buffer (190 mM Glycine, 25 mM Tris and 0.1% SDS in ddH_2_0, adjusted to a pH of 8.3) at 120 V for 90 min for protein separation via electrophoresis. Separated proteins were transferred to a PVDF 0.2 μm membrane (Amersham Hybond, GE Healthcare Life Sciences) in 1× transfer buffer (48 mM Tris, 39 mM glycine, 20% methanol and 0.0375% SDS), which was activated with 100% methanol. Wet transfer was achieved overnight at 40 V. After transfer, the membrane was blocked in 5 ml blocking buffer (2% BSA in 0.2% PBS-TWEEN) for 30 min at room temperature. Membranes were incubated overnight at 4 °C in the primary antibody cocktail (1:1000 monoclonal LC3B/MAP1LC3B antibody produced in rabbit in 0.2% PBS-Tween, 2% BSA) purchased from Novus Biological [Littleton, Colorado (CO), USA]. Membranes were washed three times in washing buffer for 15 min each. This was followed by 1 h incubation with the secondary antibody (anti-rabbit IgG antibody raised in goat labelled with horse-radish peroxidase (HRP) (1:10,000) (KPL, Mayland, USA) in 2.5% BSA in PBS. Membranes were washed three times in washing buffer for 15 min each. Pierce^®^ ECL western blotting reagent (Thermo Fisher Scientific, MA, USA) was used to activate HRP activity to visualize proteins using ChemiDoc MP (Bio-Rad, CA, USA). Monoclonal anti-actin antibody produced in mouse (1:5000, Sigma-Aldrich, St. Louis, USA) was used to standardise the membranes and developed using the anti-mouse IgG secondary antibody raised in goat labelled with HRP (KPL, Mayland, USA). Image Lab 5.2.1 (Bio-Rad, CA, USA) was used to determine band size. Three biological repeats were done.

### Statistical analysis

Morphology studies supplied qualitative data and crystal violet staining and flow cytometry analysis supplied quantitative data. Flow cytometry was repeated 3 times and involved the analysis of at least 10,000 cells per run. Three independent experiments were performed and data were shown as the mean ± standard deviation. Analysis of variance (ANOVA) single factor model of significance was used to statistically analyze data and followed by a two-tailed Student’s *t*-test. A statistical significant *P* value of <0.05 was used and indicated with an asterisk (*) and (#) was used to indicate a statistically significant difference between ESE-15-ol-exposed cells and those exposed in the presence of 3MA. Means are presented with bar-graphs with T-bars referring to standard deviations.

## Results

### Inhibition of autophagy decreases the cytotoxicity of ESE-15-ol as determined by spectrophotometry

**Fig. 2:**
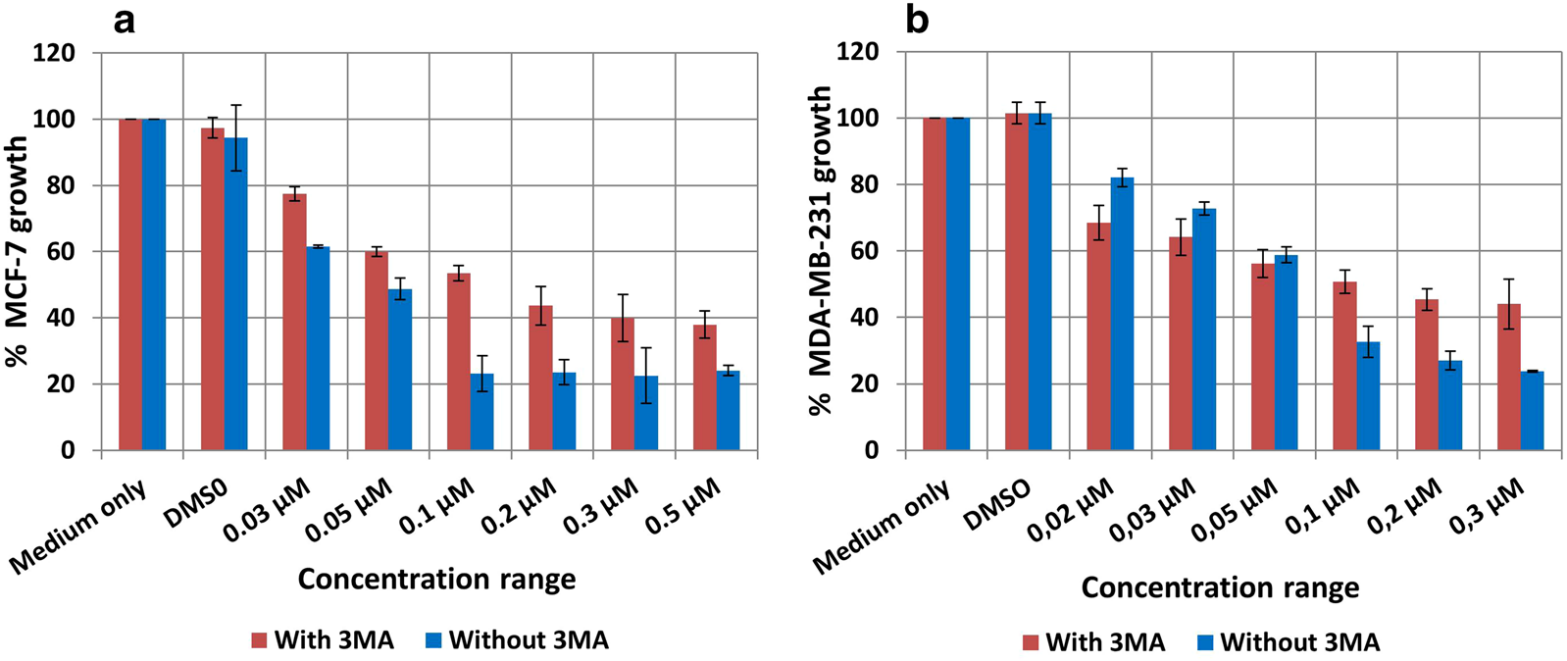
Cytotoxicity study for ESE-15-ol with/without 3MA over a 24 h exposure period in MCF-7 and MDA-MB-231 breast cancer cells. **a** The dose dependent curve for MCF-7 cells showed an IG_50_ of 0.15 μM for ESE-15-ol with 3MA and 0.05 μM for ESE-15-ol only (*P*-value = 0.007); **b** MDA-MB-231 cells showed an IG_50_ of 0.13 μM for ESE-15-ol with 3MA and 0.065 μM for ESE-15-ol only (*P*-value = 0.0195). *Bars* indicate averages of three independent biological repeats, each with n = 3. *Error bars* represent standard deviation

Spectrophotometry was used to determine the effect of autophagy inhibition on the cytotoxicity of ESE-15-ol-exposed human adenocarcinoma breast cancer cells (MCF-7) and metastatic human adenocarcinoma breast cancer cells (MDA-MB-231). The half maximum growth inhibitory concentrations (IG_50_) of ESE-15-ol with and without 3MA were determined by dose-dependent studies over a 24 h period. The IG_50_ of ESE-15-ol was calculated at 0.05 ± 0.018 μM and at 0.15 ± 0.014 μM for ESE-15-ol with 3MA in MCF-7 cells (Fig. 2a). The IG_50_ of ESE-15-ol was calculated at 0.065 ± 0.005 μM, and at 0.13 ± 0.06 μM for ESE-15-ol with 3MA-exposed MDA-MB-231 cells (Fig. 2b). Autophagy inhibition was thus seen to have caused a statistically significant decrease in ESE-15-ol cytotoxicity, with a *P* value of 0.007 in MCF-7 cells and 0.0195 in MDA-MB-231 cells.

### Morphological features of cell death induced by ESE-15-ol were atteniated by addition of 3MA

**Fig. 3:**
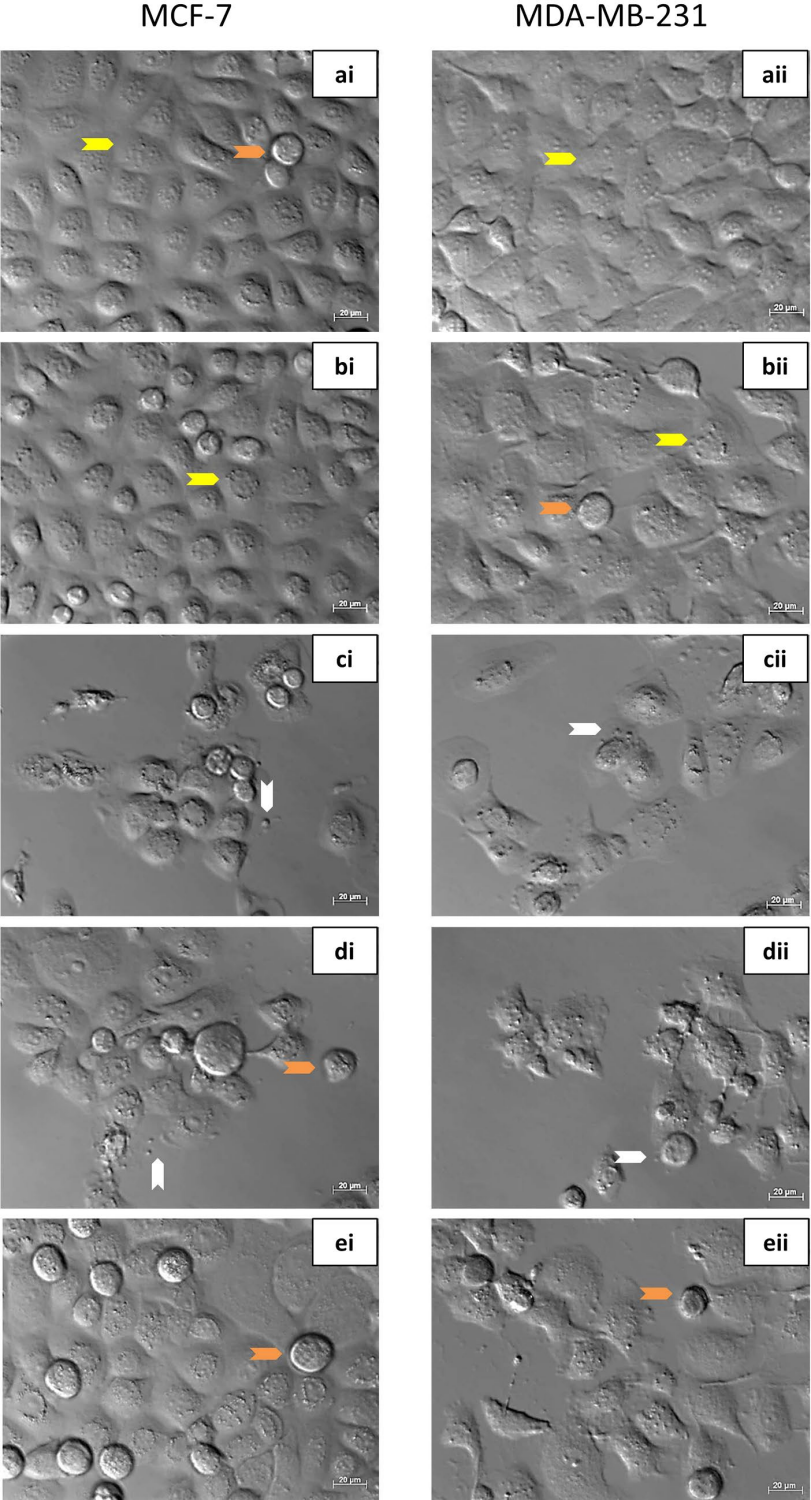
PlasDIC images of MCF-7 and MDA-MB-231 cells exposed to the compound with/or without 3MA for 24 h. **i** MCF-7 cells and **ii** MDA-MB-231 cells grown in **a** DMSO and **b** 3MA served as negative controls. Confluent cell growth with no signs of cell distress was demonstrated. **c** Actinomycin D (0.1 μg/ml) served as a positive control for apoptosis, resulting in apoptotic body formation and compromised cell density. **d** ESE-15-ol-treated cells revealed the presence of rounded cells, formation of apoptotic bodies and decreased cell density. **e** ESE-15-ol exposure to cells in which autophagy had been inhibited with 3MA showed an increase in cell viability. (Arrow colour key: *Yellow* = interphase cells; *orange* = rounded cells in metaphase; *white* = apoptotic bodies.)

Polarization-optical transmitted light differential interference light microscopy (PlasDIC) was used to evaluate the morphological response of cells to ESE-15-ol with or without 3MA. MCF-7 (Fig. 3ai) and MDA-MB-231 (Fig. 3aii) cells exposed to DMSO showed no signs of cell distress. Confluent cell growth was seen with visible nucleoli as for the 3MA-exposed cells (Fig. 3bi, bii). Cells were mostly present in interphase. Actinomycin D-treated cells showed a decrease in cell density for both MCF-7 (Fig. 3ci) and MDA-MB-231 (Fig. 3cii) cells. Apoptotic body formation, cell debris and shrunken cells were visible, which are characteristic of apoptotic cell death. ESE-15-ol-treated MCF-7 (Fig. 3di) and MDA-MB-231 (Fig. 3dii) cells demonstrated an increased proportion of rounded cells as well as the presence of apoptotic bodies. ESE-15-ol-treated cells together with 3MA showed apoptotic body formation and rounded cells in both MCF-7 (Fig. 3ei) and MDA-MB-231 cells (Fig. 3eii), but to a lesser extend when compared to cells treated with ESE-15-ol without 3MA.

### Acidic vacuoles were decreased in cells treated with ESE-15-ol in combination with 3MA

**Fig. 4:**
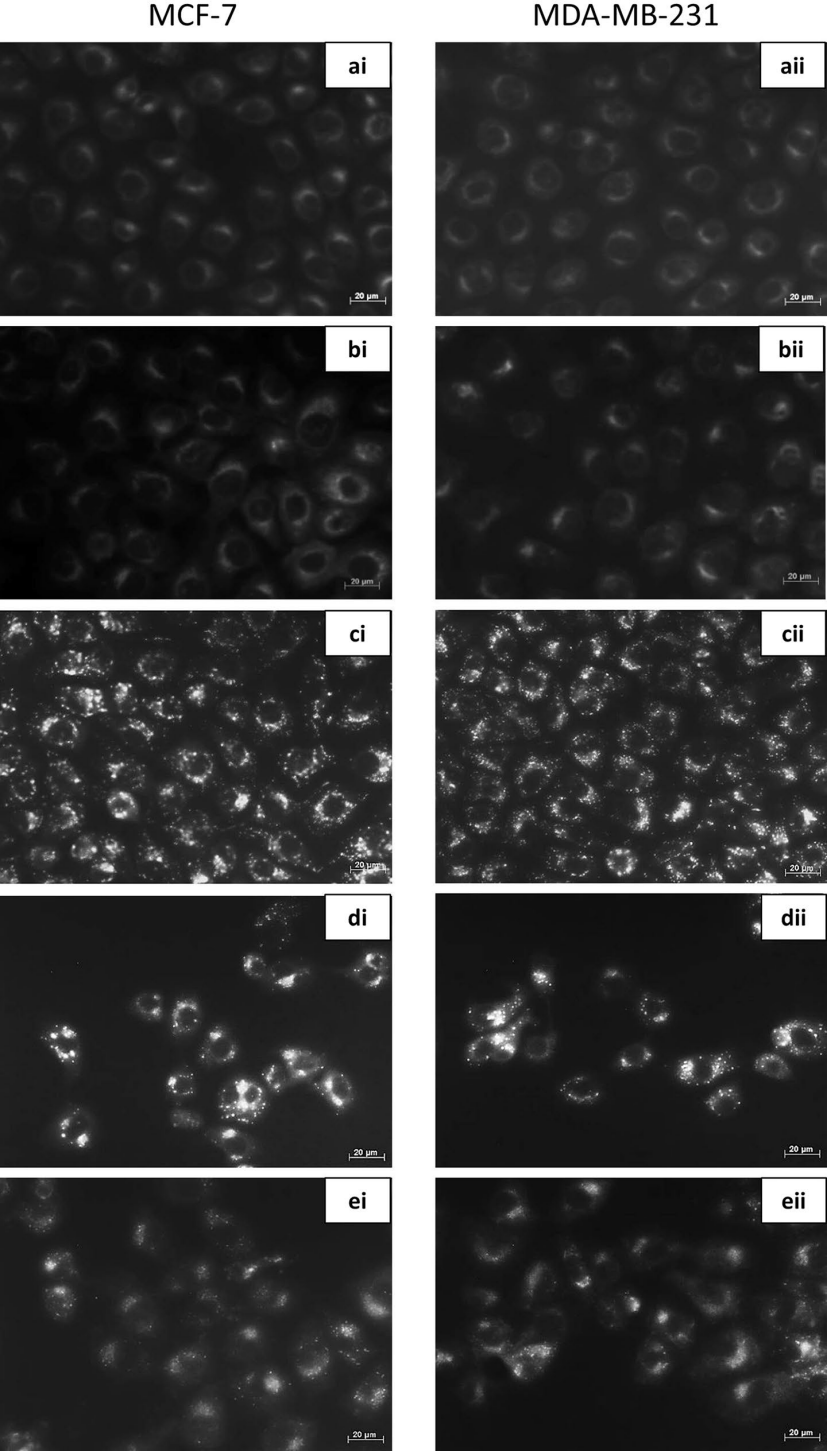
Fluorescent microscopy with monodansylcadaverine staining of MCF-7 and MDA-MB-231 cells. **i** MCF-7 and **ii** MDA-MB-231 cells treated with **a** DMSO and **b** 3MA served as negative controls and displayed non-specific MDC staining. **c** Tamoxifen was used as a positive control for acidic vacuoles and displayed clear MDC stained vacuoles. **d** ESE-15-ol treated cells showed increased MDC staining, while **e** ESE-15-ol treated cells together with 3MA showed less distinctive MDC staining, indicating partial autophagy inhibition (40× magnification)

Monodansylcadaverine (MDC) is a weak base fluorescent stain which stains acidic vacuoles that suggest occurence of autophagy [34]. MCF-7 and MDA-MB-231 cells exposed to DMSO (Fig. 4ai, aii) and 3MA (Fig. 4bi, bii) showed non-specific MDC staining. Both controls displayed confluent cell growth. Tamoxifen-treated cells (Fig. 4ci, cii) showed an increase in MDC-stained vacuoles in both cell lines. ESE-15-ol-treated cells demonstrated an increase in MDC-stained vacuoles, as well as decreased cell density (Fig. 4di, dii). ESE-15-ol treated cells in the presence of 3MA showed a decrease in MDC staining, with less acidic vacuole formation apparent when compared to the drug treated sample (Fig. 4ei, eii).

### Vacuole formation in response to ESE-15-ol exposure was evident but diminshed when co-incubated with 3MA

**Fig. 5:**
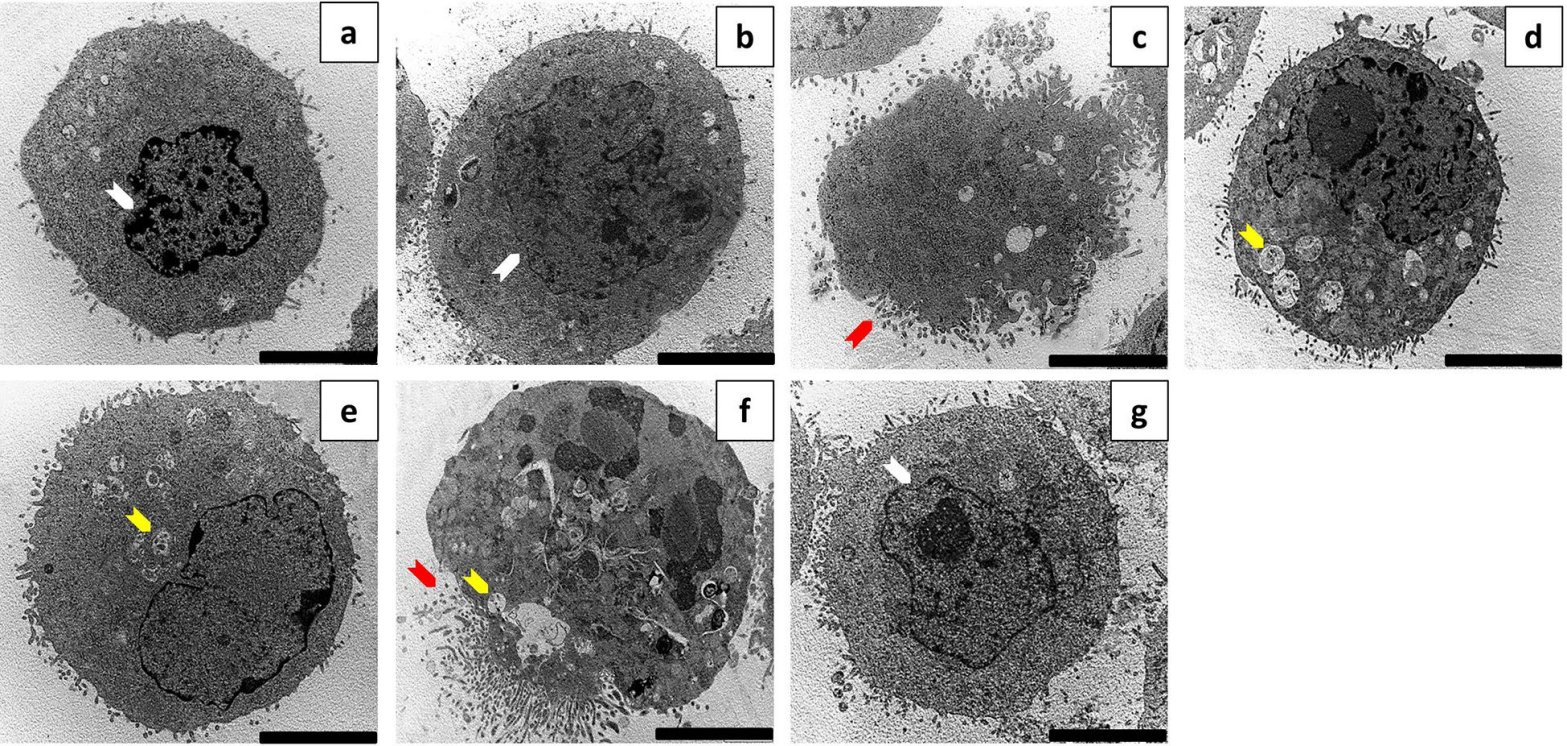
Transmission electron micrographs of MCF-7 cells. MCF-7 cells treated with **a** DMSO and **b** 3MA showed intact nuclear envelope and cytoplasmic membranes with minimal cell protrusions. **c** Actinomycin D-exposed cells showed membrane blebbing and apoptotic body formation. **d** Tamoxifen-treated cells showed an increase in vesicle formation when compared to **e** tamoxifen with 3MA. **f** ESE-15-ol-treated cells showed hypercondensed chromatin with an increase in vesicle- and apoptotic body formation. **g** Cells exposed to ESE-15-ol concurrent to autophagy inhibition with 3MA showed less signs of cell distress, fewer vesicles and intact nuclear membranes (*Scale bar* 5 μM) (Arrow colour keys: *white* = intact nuclear membrane; *red* = apoptotic bodies; *yellow* = vesicles)

**Fig. 6:**
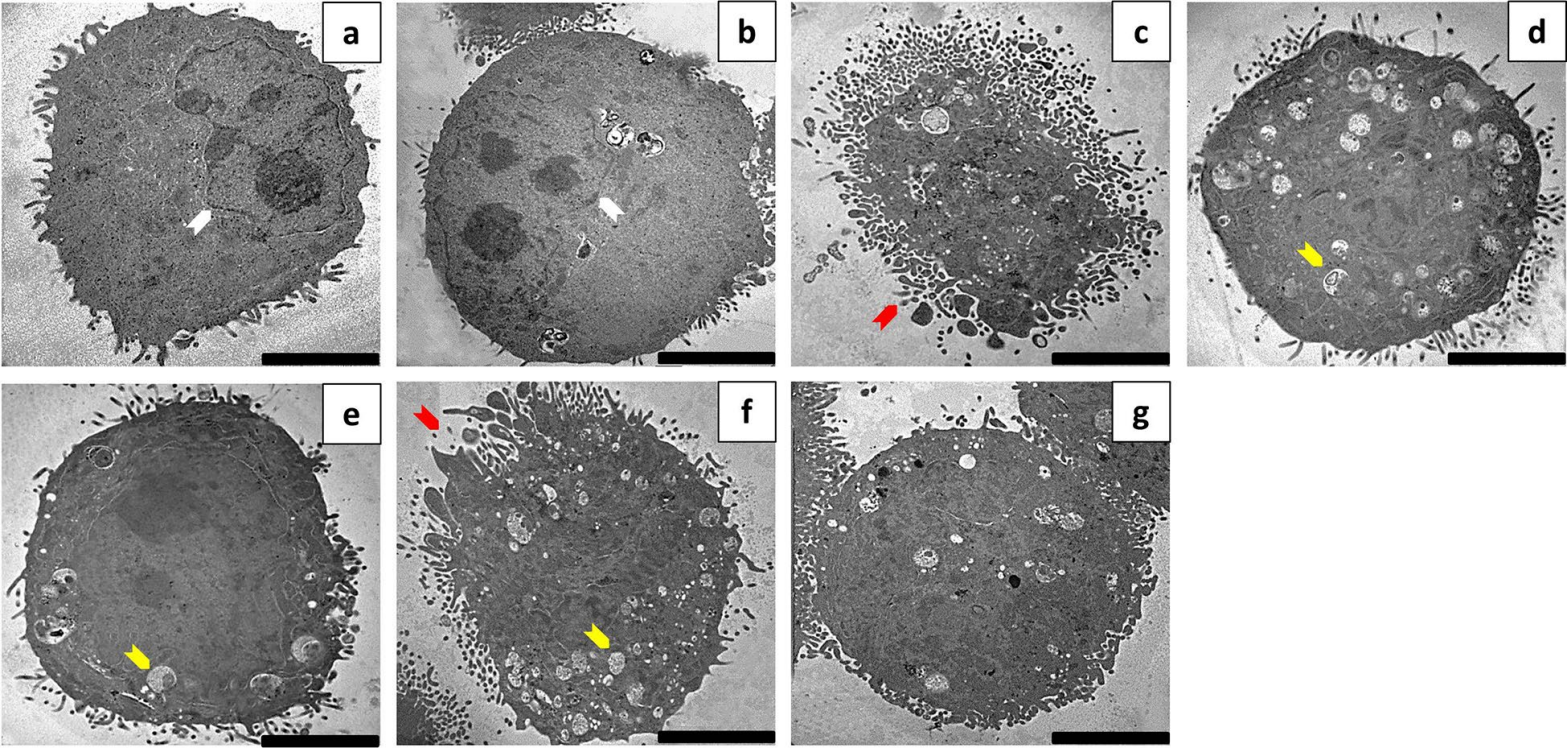
Transmission electron micrographs of MDA-MB-231 cells. MDA-MB-231 cells treated with **a** DMSO and **b** 3MA showed an intact nuclear membrane and well-defined cell membranes with minimal cell protrusions. **c** Actinomycin D-exposed cells showed membrane blebbing and apoptotic body formation. **d** Tamoxifen-treated cells showed more vesicle formation when compared to cells treated with **e** tamoxifen with 3MA. **f** ESE-15-ol-treated cells showed hyper condensed chromatin with increased vesicles and apoptotic body formation. **g** Cells exposed to ESE-15-ol with 3MA showed fewer vesicles with an intact nuclear membrane (*Scale bar* 5 μM) (Arrow colour keys: *white* = intact nuclear membrane; *red* = apoptotic bodies; *yellow* = vesicles)

Transmission electron microscopy was used to analyze the ultrastructure of ESE-15-ol-treated MCF-7 and MDA-MB-231 cells, with and without autophagy inhibition by 3MA. Cells propagated in DMSO (Figs. 5a, 6a) showed a smooth cell membrane with minimal cellular protrusions, together with an intact nuclear envelope. No morphological differences were seen between cells exposed to DMSO and 3MA (Figs. 5b, 6b). Cells treated with actinomycin D (Figs. 5c, 6c) increased membrane blebbing and apoptotic body formation representative of apoptotic cell death. Tamoxifen-treated cells showed an increase in vesicle formation (Figs. 5d, 6d). Less vacuole formation was observed in tamoxifen-treated cells with 3MA (Figs. 5e, 6e). This suggests partial autophagy inhibition by 3MA. Intact cell membranes were observed in tamoxifen-treated cells, with and without 3MA. ESE-15-ol-treated cells (Figs. 5f, 6f) displayed an increase in vacuolar structures indicative of autophagy. Apoptotic bodies, hypercondensed chromatin and increased cell protrusions were also observed in cells exposed to ESE-15-ol. ESE-15-ol-treated cells with 3MA inhibition of autophagy (Figs. 5g, 6g) revealed cells with intact nuclear- and cell-membranes. A decrease in cellular distress with fewer vesicles was observed in these cells.

#### 3MA co-incubation increased cell viability and reduced G_2_/M block as well as the sub-G_1_ population in response to ESE-15-ol exposure

**Fig. 7:**
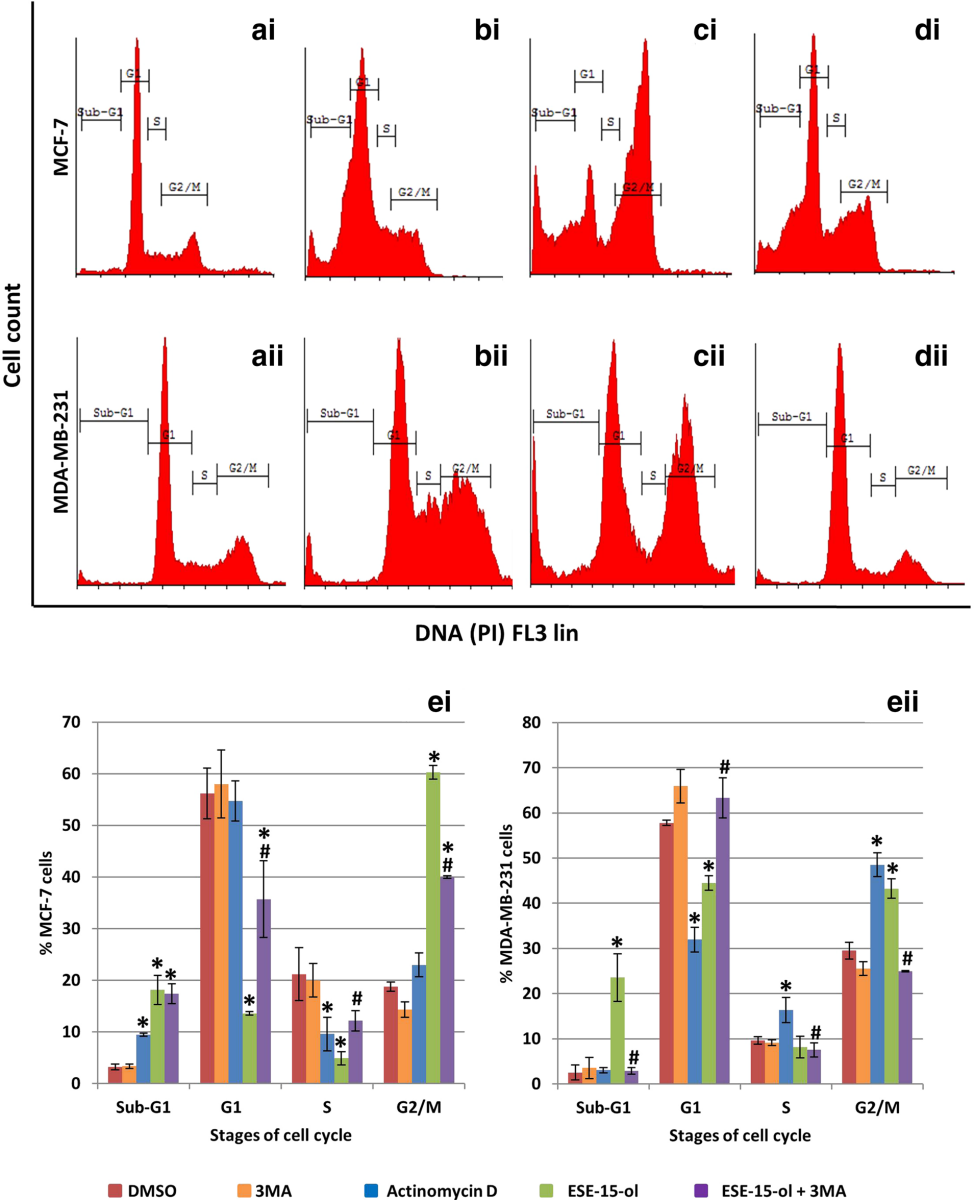
Cell cycle analysis of MCF-7 and MDA-MB231 cells exposed to ESE-15-ol with- and without 3MA. Cells were exposed to DMSO as a negative vehicle control (**ai**, **aii**) which showed a prominent G_1_ phase. Actinomycin D (**bi**, **bii**) was used as a positive control for apoptosis which resulted in an increase in the sub-G_1_ phase. An increase in the G_2_/M phase was seen in ESE-15-ol-treated cells (**ci, cii**) with a concurrent decrease in the G_1_ phase. ESE-15-ol treated cells together with 3MA (**di**, **dii**) showed a decrease in the G_2_/M phase with an increase in the G_1_ phase. Graphical representation of **ei** MCF-7 and **eii** MDA-MB-231 cell cycle analysis. ESE-15-ol-treated cells together with 3MA demonstrated an increase in the G_1_ phase when compared to ESE-15-ol-treated cells (*P* value <0.05; standard deviation represented by* T-bars*; *indicates statistical difference between compounds and DMSO vehicle control; ^#^indicates statistical difference between ESE-15-ol and ESE-15-ol with 3MA)

The quantification of cells at various stages within the cell cycle was determined with flow cytometry. Cell cycle distribution of MCF-7 cells exposed to DMSO (Fig. 7ai) showed an average of 3.24 ± 0.54% in the sub-G_1_ phase, 56.2 ± 4.89% in the G_1_ phase and 18.79 ± 0.89% in the G_2_/M phase. Cell cycle distribution of MDA-MB-231 cells grown in DMSO (Fig. 7aii) showed an average of 2.53 ± 1.62% in the sub-G_1_ phase, 57.8 ± 0.61% in the G_1_ phase and 29.5 ± 1.83% in the G_2_/M phase. No statistical significance was found between cells grown in medium only and treated with DMSO or 3MA, indicating that the vehicle was non-toxic to the cells. MCF-7 and MDA-MB-231 cells exposed to actinomycin D and ESE-15-ol had statistically significant changes in their cell cycle distributions when compared to the DMSO vehicle control. Actinomycin D-treated MCF-7 cells (Fig. 7bi) had a significant increase in the sub-G_1_ phase (9.47 ± 0.31%; *P* = 0.00015) and the G_2_/M (22.98 ± 2.32%; *P* = 0.04) phase. ESE-15-ol-treated MCF-7 cells (Fig. 7ci) had a significant increase in the sub-G_1_ phase (18.14 ± 2.81%; *P* = 0.001) and the G_2_/M phase (60.28 ± 1.34%; *P* = 0.0009) when compared to the DMSO control. ESE-15-ol-treated MCF-7 cells together with 3MA autophagy inhibition (Fig. 7di) had a statistically significant increase in the G_1_ phase (35.70 ± 7.45%; *P* = 0.02) when compared to ESE-15-ol without 3MA treatment (13.59 ± 0.36%). Actinomycin D treated MDA-MB-231 cells (Fig. 7bii) had a significant decrease in the G_1_ phase (31.94 ± 2.75%; *P* = 0.0004) and concomitant increase in the G_2_/M phase (48.52 ± 2.63%; *P* = 0.02) when compared to DMSO-exposed cells. ESE-15-ol-treated MDA-MB-231 cells (Fig. 7cii) had a significant decrease in the G_1_ phase (44.45 ± 1.59%; *P* = 0.008) with a significant increase in the G_2_/M phase (43.23 ± 2.13%; *P* = 0.009) when compared to DMSO vehicle control. ESE-15-ol-treated MDA-MB-231 cells with a concurrent treatment with 3MA (Fig. 7dii) had a statistically significant increase in the G_1_ phase (63.64 ± 4.46%; *P* 0.03) when compared to cells exposed to ESE-15-ol without 3MA. These results indicate that autophagy inhibition decreases the cytotoxic effect of ESE-15-ol exposure in MCF-7 and MDA-MB-231 breast cancer cells.

### Apoptosis induction in response to ESE-15-ol was diminshed with addition of 3MA

**Fig. 8:**
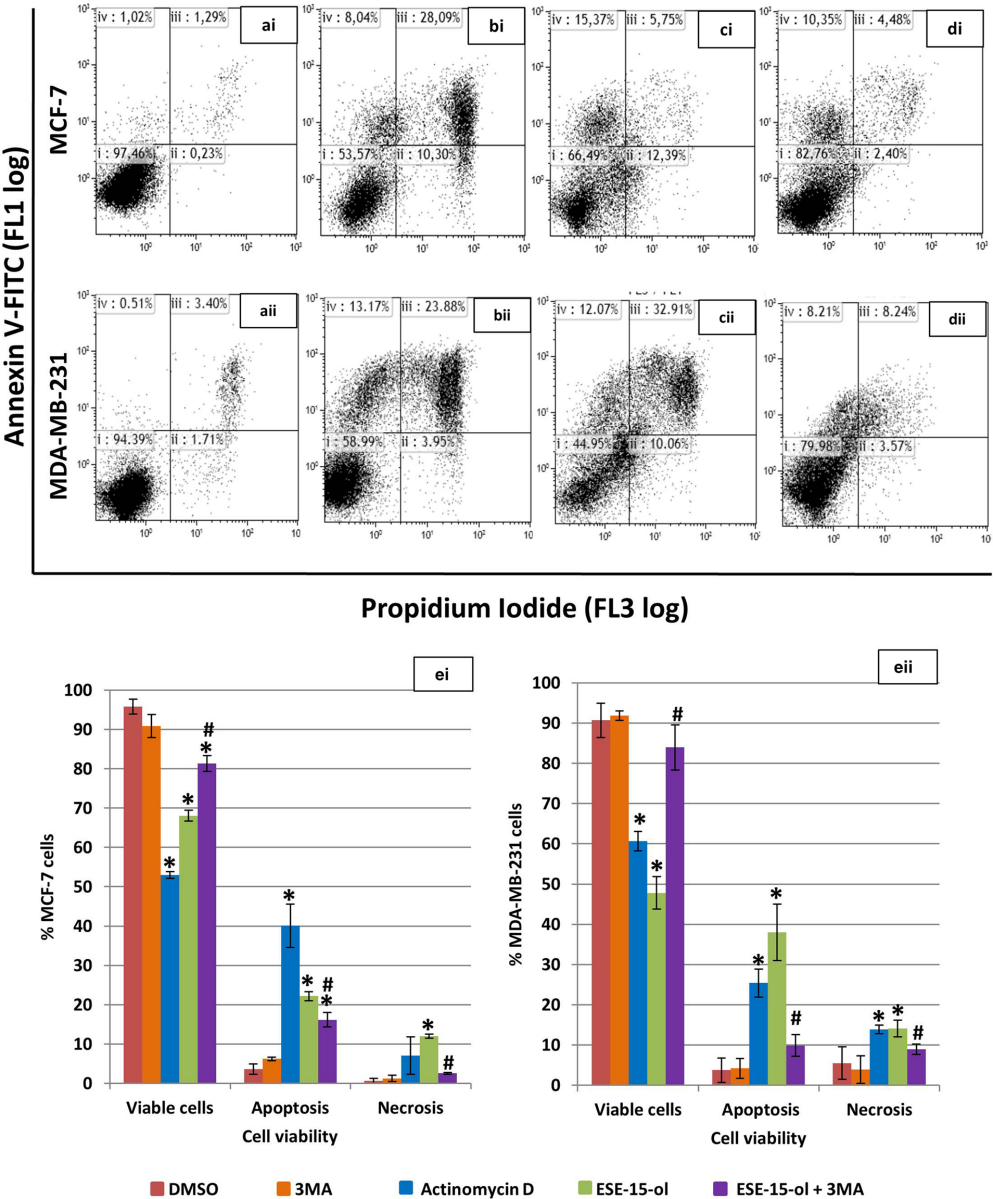
Annexin-V FITC flow cytometric analysis of cell viability. Dot blots of **i** MCF-7 and **ii** MDA-MB-231 cells exposed to **a** DMSO as a vehicle control and demonstrated a viable cell population. *Dot blots* of **b** actinomycin D (positive apoptosis control) and **c** ESE-15-ol-treated cells showed increased cell death via apoptosis. **d** ESE-15-ol-treated cells with 3MA showed increased cell viability when compared to cells exposed to ESE-15-ol only. Graphical representation of (ei) MCF-7 and (eii) MDA-MB-231 cells showed a decrease in cell viability in cells treated with ESE-15-ol when compared to DMSO. An increase in viable cells is observed in ESE-15-ol treated cells with 3MA when compared to ESE-15-ol treated cells. *Bars* represent averages of three biological repeats (*P* value <0.05; standard deviations represented by* T-bars*; *indicates statistical difference between compounds and DMSO vehicle control; ^#^indicates statistical difference between ESE-15-ol and ESE-15-ol with 3MA) (Dot plot keys: **i** = viable cells; **ii** = necrosis; **iii** = late apoptosis; **iv** early apoptosis)

Flow cytometry employing annexin V-FITC and PI was used to distinguish between viable-, apoptotic- and necrotic cells. Statistical analysis of dot plots data indicated a statistically significant increase in apoptosis in actinomycin D-treated (Fig. 8bi) (40.06 ± 5.55%; *P* = 0.001) and ESE-15-ol-treated (Fig. 8ci) (21.21 ± 0.13%; *P* = 0.002) MCF-7 cells when compared to the DMSO-vehicle control (Fig. 8ai). A statistically significant increase in apoptosis in actinomycin D-treated (Fig. 8bii) (25.39 ± 3.44%; *P* = 0.0006) and ESE-15-ol-treated (Fig. 8cii) MDA-MB-231 cells (38 ± 7.02%; *P* = 0.004) was detected when compared to the DMSO-vehicle control (Fig. 8aii). No statistically significant difference in viability was detected between cells grown in medium only, DMSO-exposure and 3MA-exposure in both MCF-7 and MDA-MB-231 cells. A statistically significant increase in viability in ESE-15-ol-treated MCF-7 and MDA-MB-231 cells together with 3MA (81.31 ± 2.05%; *P* = 0.01 and 83.97 ± 5.6%; *P* = 0.01, respectively) was observed when compared to ESE-15-ol-treated cells (Fig. 8ei, eii).

### LC3 expression was increased in cells exposed to ESE-15-ol

**Fig. 9:**
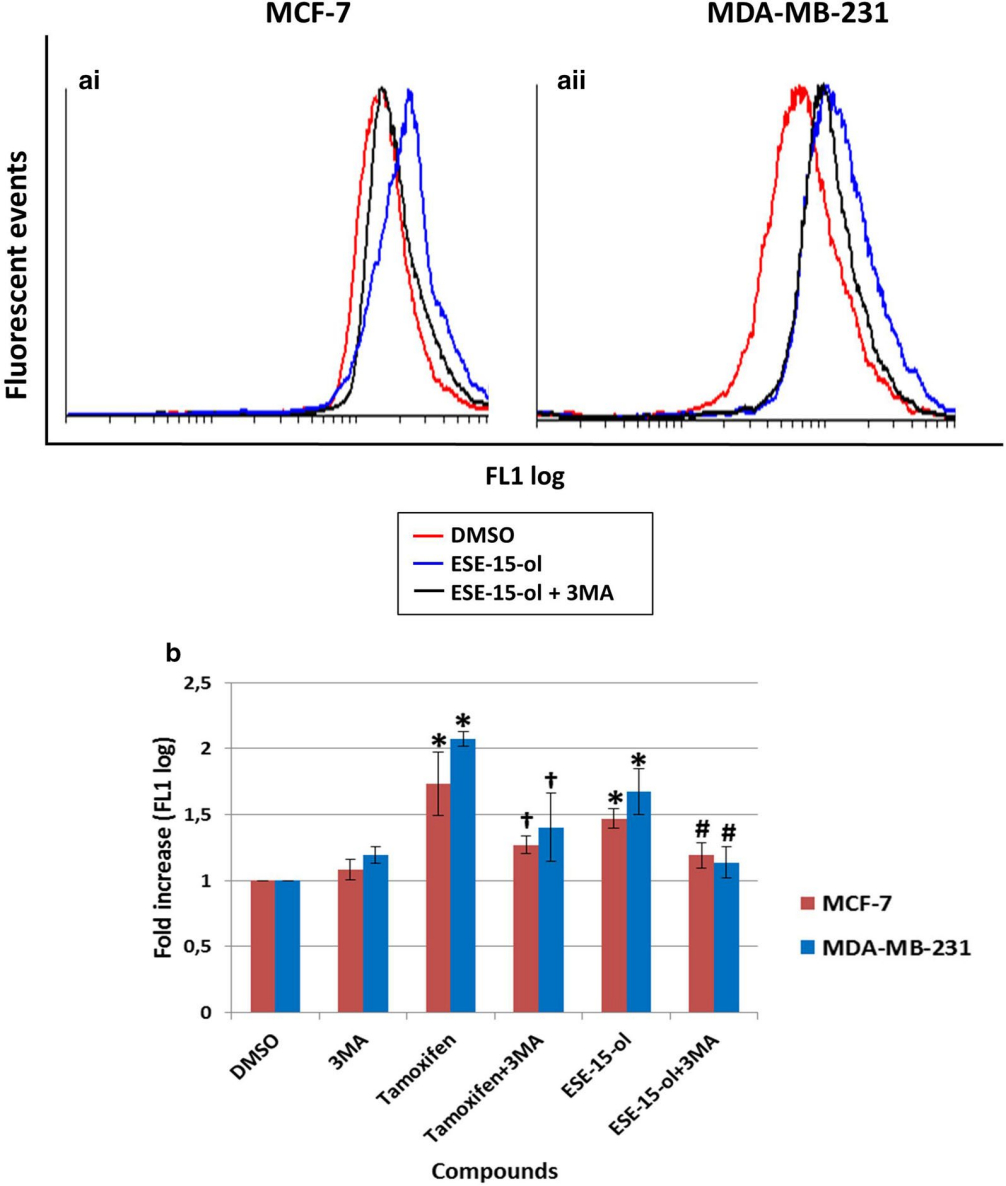
LC3 fluorescence determination within MCF-7 and MDA-MB-231 cells after a 24 h exposure. Overlay histogram of **ai** MCF-7 and **aii** MDA-MB-231 exposed cells. Cells treated with ESE-15-ol showed a right shift which was greater than ESE-15-ol with 3MA. **b** Graphical representation shows an increase in LC3 detection within ESE-15-ol-treated cells. *Bars* represent averages of three biological repeats (*P* value <0.05; standard deviations represented by* T-bars*; *indicates statistical differences between compounds and DMSO vehicle control; ^#^indicates statistical difference between ESE-15-ol and ESE-15-ol with 3MA; ^†^indicates statistical difference between tamoxifen and tamoxifen with 3MA)

Flow cytometry employing a microtubule-associated protein 1A/1B-light chain 3 (LC3) conjugated rabbit polyclonal anti-LC3B antibody (Novus Biologicals, Littleton, CO) was used to quantify and confirm induction and inhibition of autophagy. Figure 9a illustrates an overlay histogram of MCF-7 (1) and MDA-MB-231(2) cells exposed to ESE-15-ol with and without 3MA. A right shift is seen in ESE-15-ol-treated cells without 3MA. A decrease in LC3 detection is seen in cells exposed to ESE-15-ol together with 3MA indicating partial autophagy inhibition. No difference was detected between cells grown in medium only and cells exposed to the DMSO vehicle control and 3MA (not shown in overlay). Graphical representations in Fig. 9b show a statistically significant fold increase of 2.075 ± 0.05 LC3 after tamoxifen exposure and 1.67 ± 0.17 LC3 in ESE-15-ol when compared to the DMSO vehicle control. A statistically significant fold decrease of LC3 is seen after tamoxifen with 3MA exposure (1.40 ± 0.25; *P* = 0.022) when compared to tamoxifen exposure alone, as well as between ESE-15-ol with 3MA (1.13 ± 0.11; *P* = 0.004) when compared to ESE-15-ol alone.

**Fig. 10:**
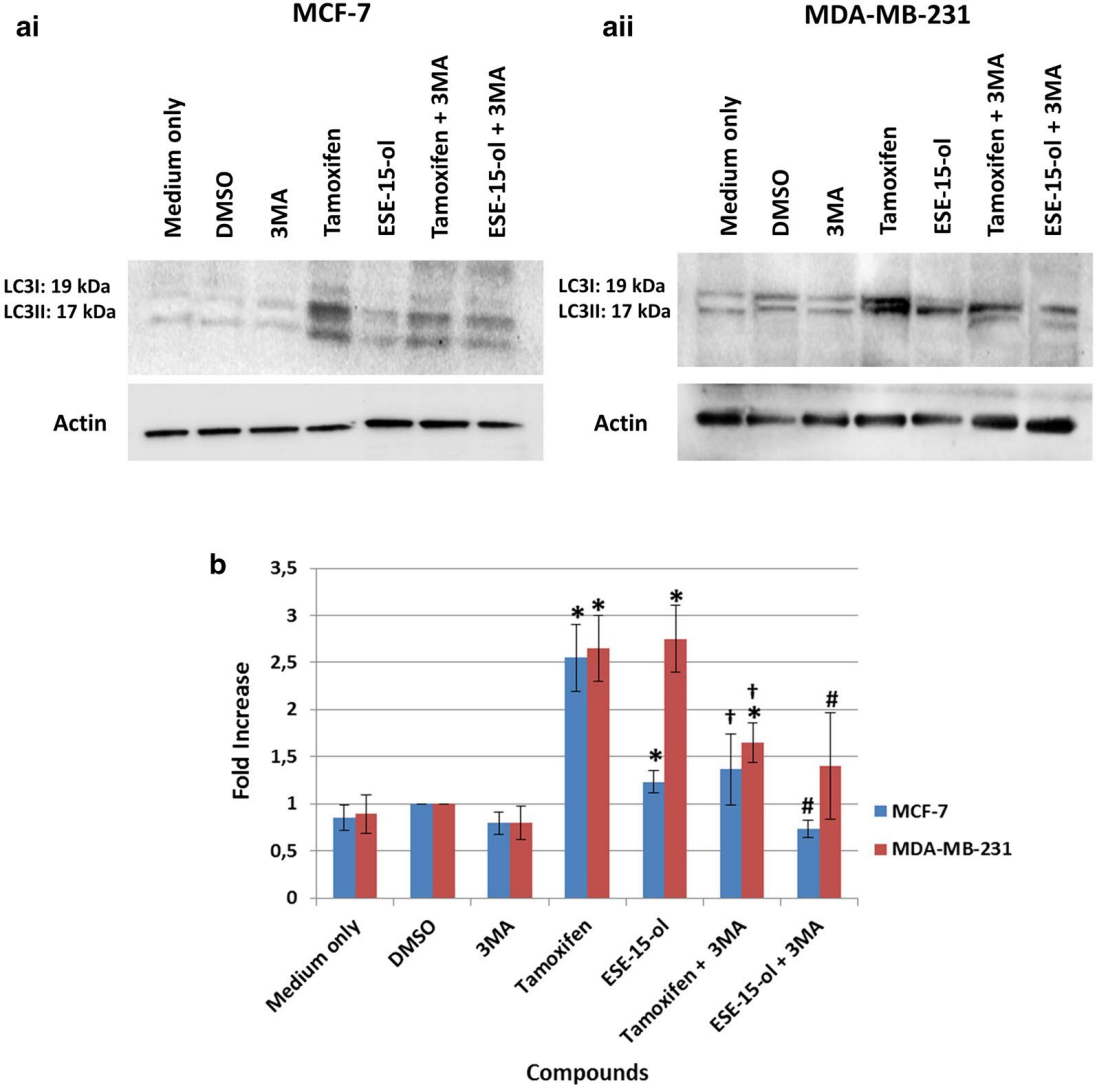
Western blot analysis of LC3II protein expression levels. Western blots of **ai** MCF-7 and **aii** MDA-MB-231 cells treated with ESE-15-ol showed a more prominent LC3II band than cells treated with ES-15-ol with 3MA. **b** Graphical representation demonstrates a statistically significant decrease in LC3II protein levels within cells exposed to ESE-15-ol concurrently with 3MA as well as tamoxifen with 3MA. Bars represent averages of three biological repeats (*P* value <0.05; standard deviations represented by* T-bars*; *indicates statistical differences between compounds and DMSO vehicle control; ^#^indicates statistical difference between ESE-15-ol and ESE-15-ol with 3MA; ^†^indicates statistical difference between tamoxifen and tamoxifen with 3MA)

### LC3I conversion to LC3II decreased in cells treated with ESE-15-ol together with 3MA

LC3II has a role in cytoplasmic cargo selection, autophagosome membrane tethering and fusion [35]. Increased levels of LC3-II may be associated with an up-regulation in autophagy or a decrease in turnover (altered autophagic flux). Western blots were used to quantify the formation of LC3II in the presence of the analogue with- and without 3MA (Fig. 10). Protein quantification revealed a statistically significant fold-increase in LC3II expression when MCF-7 cells were exposed to ESE-15-ol (1.23 ± 0.11, *P* = 0.02) and tamoxifen (2.55 ± 0.35, *P* = 0.003, as did the MDA-MB-231 cells (2.75 ± 0.35, *P* = 0.002 and 2.4 ± 0.42, *P* = 0.004 respectively), when compared to the DMSO control. Addition of 3MA reduced LC3II expression to 0.73 ± 0.09-fold (MCF-7 cells) and to 1.4 ± 0.56 (MDA-MB-231 cells), statistically significant when compared to ESE-15-ol only-exposed MCF-7 (*P* = 0.015) and MDA-MB-231 cells (*P* = 0.04) respectively. Similar trends were seen in the tamoxifen-treated positive control samples.

## Discussion

2ME is an anti-proliferative agent which shows promise in cancer treatment, including breast cancer [36]. It induces both the intrinsic- and extrinsic apoptotic pathways through inactivation of Bcl-2 and increased DR5 expression [5]. Several promising analogues of 2ME, such as 2-methoxyestradiol-bis-sulfamate (2MEOE2bisMATE) have been developed for improved potency and bioavailability of the parent drug [9, 36, 37]. Our laboratory has in silico-designed a range of sulphamoylated estrone analogues with the added intention to localise them to solid tumour micromilieus through an increased CAIX binding affinity [9, 38].

Of these compounds, ESE-15-ol, 2-ethyl-3-*O*-sulphamoyl-etsra-1,3,5 (10) 15-tetraene-3-ol-17-one (ESE-15-one) and 2-ethyl-3-*O*-sulphamoyl-etsra-1,3,5 (10) 16-tetraene (ESE-16) have been investigated for modes of cell death induction (apoptosis and autophagy) on various cancer cell lines [9, 36, 39–41]. Authors have indicated the increased presence of autophagic vesicles at certain time points after drug exposure [42]. Extensive analysis of the drug’s effect on induction or flux of autophagy have not been completed, although up-regulation of autophagic genes have been reported on microarrays [42]. Descriptions of autophagy conceptualize a dual role within the context of cellular survival, either inducing programmed cell death type II or activating a pro-survival phenotype [19]. Autophagy can be activated by tumor microenvironments to protect hypoxic and nutrient deprived cells, thus inducing resistance to drug treatment [43]. The current study aimed to determine the role of autophagy associated demonstrated on cancer cell exposure to ESE-15-ol by inhibiting autophagy with 3MA.

Cytotoxicity studies on MCF-7 and MDA-MB-231 breast adenocarcinoma cells were conducted over a 24 h period by spectrophotometric quantification of crystal violet. IG_50_ values were determined for ESE-15-ol-treated cells, and compared to the value obtained when 5 mM 3MA, an inhibitor of autophagy, was added for the duration of the drug exposure. This was done to determine the effect which autophagy inhibition would have on the cytotoxicity of ESE-15-ol on MCF-7 and MDA-MB-231 cells. IG_50_ values of 0.05 and 0.065 μM were calculated for MCF-7 and MDA-MB-231 cells respectively when exposed to ESE-15-ol without 3MA. IG_50_ values of 0.15 and 0.1 μM in MCF-7 and MDA-MB-231 cells respectively were determined for ESE-15-ol together with 3MA. The increase in IG_50_ values with the addition of 3MA were statistically significant in both cell lines (*P* = 0.007; *P* = 0.0195). ESE-15-ol without 3MA had an increased cytotoxicity than with 3MA. The results indicate the possibility that autophagy contributes to the cell death process in cells exposed to ESE-15-ol.

Various microscopic techniques were used to determine the effect of autophagy inhibition on the morphology of MCF-7 and MDA-MB-231 cells after ESE-15-ol-exposure. PlasDIC microscopy revealed that ESE-15-ol-exposure resulted in numerous rounded cells, most likely in mitotic arrest, supporting its action as a spindle poison. Cell cycle analysis corroborated this induction due to an increase in cells present in the G_2_/M phase. Previous studies have reported that a mitotic block was caused in MCF-7 cells exposed to 1 μM 2ME, the parent compound of ESE-15-ol [5]. Apoptotic bodies and shrunken cells were visualized on the micrographs of ESE-15-ol-exposed cells. This suggests that ESE-15-ol induced cell death via apoptosis over a 24 h exposure period due to spindle disruption. Theron et al. [11] showed that ESE-16-ol and ESE-15-one induced metaphase block due to abrogation of microtubule dynamics and non-satisfaction of the spindle assembly checkpoint (SAC), thereby inducing apoptosis in MDA-MB-231 and HeLa cells. In this study, only a small proportion of cells demonstrated a mitotic arrest, although apoptotic bodies were still visible when cells were treated with ESE-15-ol combined with 3MA (an effect which was more subtle when compared to cells exposed to the compound-only). Thus signs of apoptotic cell death were still present when autophagy was inhibited. For optimal cell death by ESE-15-ol both apoptosis and autophagy are hypothesized to be involved.

Fluorescent microscopy employing MDC was used to detect acidic vacuoles that may be indicative of autophagy. MCF-7 and MDA-MB-231 cells treated with ESE-15-ol over a 24 h period showed positive staining for MDC, with more discrete vacuolar formation. Cells treated with ESE-15-ol in combination with 3MA showed a decrease in MDC staining when compared to cells treated with ESE-15-ol alone. This indicates that 3MA blocked autophagy induced by ESE-15-ol, albeit incompletely. TEM was conducted to investigate the effects of ESE-15-ol as well as, autophagy inhibition on the ultrastructure of MCF-7 and MDA-MB-231 cells. Cells exposed to ESE-15-ol and tamoxifen (positive control) showed an increased number of vacuoles which are indicative autophagic vacuoles. Apoptotic bodies were observed in ESE-15-ol-treated cells, together with cellular distress, chromatin hypercondensation and membrane blebbing, indicating cell death via apoptosis. Cells exposed to ESE-15-ol together with 3MA demonstrated significantly less distress and a decrease in vacuole formation.

Apoptotic characteristics observed in the morphology studies of the drug-exposed cells were quantified using flow cytometry. Analysis of cell cycle progression showed a statistically significant decrease in mitotic arrest when MCF-7 and MDA-MB-231 cells were treated with ESE-15-ol with 3MA when compared to ESE-15-ol without 3MA. A statistically significant increase in the G_1_ phase was also demonstrated when cells were treated with ESE-15-ol with 3MA when compared to ESE-15-ol without 3MA. Quantification of the translocation of PS to the outer membrane of the cell during apoptosis was also done by flow cytometry. ESE-15-ol-exposed cells showed an increase in apoptosis to 21.21% for MCF-7 cells and 38% for MDA-MB-231 cells with a decrease in cell viability. Autophagy inhibition demonstrated a decrease in apoptosis (16.13% for MCF-7 cells and 6.74% for MDA-MB-231 cells). This suggests that 3MA increased cell viability when combined with ESE-15-ol.

Flow cytometry studies employing LC3 detection were used to quantify the presence of autophagic vacuoles. MCF-7 and MDA-MB-231 cells treated with ESE-15-ol showed a significant fold increase in LC3 detection when compared to the DMSO vehicle control. Additionally, 3MA inhibition of autophagy were quantified and verified in tamoxifen-treated cells. The decrease of acidic vacuoles seen with MDC staining of ESE-15-ol treated cells with 3MA as well as the TEM findings were corroborated by the decrease in LC3 detection via flow cytometry. These results verified that 3MA incompletely blocked autophagy. LC3 is a marker for autophagic induction or to detect a decrease in autophagosome formation, but does however, have limitations. There are multiple isoforms of LC3 (LC3A, LC3B, LC3B2 and LC3C), as well as variation in the antibody specificity (LC3I vs LC3II). ATG8-PE/LC3II is a protein marker which detects completed autophagosome formation but does not measure or quantify autophagy flux (flow cytometry indicative of total LC3 levels and does not differentiate between LC3I and LC3II). Autophagic flux is often quantified via LC3-II turnover as detected by Western blotting [44]. When co-incubated with the autophagy inhibitor 3MA, the decreased LC3II expression in comparison to the elevated expression induced by ESE-15-ol, alludes to a disruption in autophagic flux, although these results must be interpreted with cauation due to the analytic constraints of autophagy quantification. This study indicated that the drug treatment did increase the LC3II expression, a response which was muted by co-incubation with 3MA.

Autophagosomes are transported to the microtubule-organising centre (MTOC) of cells along the microtubules where lysosome cluster are found [45]. In the MTOC, lysosome fusion and content exchange occurs after which autophagosomal degradation by lysosomal hydrolases take place [45]. It is hypothesised that ESE-15-ol increases the induction of autophagy, possibly by the increased formation of reactive oxygen species [6]. Microarray analysis of autophagy gene expression indicated up-regulation of autophagy-related genes after exposure to 17-beta estradiol analogues [42]. However, ESE-15-ol disrupts microtubule dynamics which are essential for autophagy, potentially retracting autophagosome fusion and degradation. A possible explanation for the induction of autophagy by ESE-15-ol could be the inhibition of beclin-1. 3MA can possibly increase beclin-1 expression causing autophagy inhibition. These results support previous studies done with estradiol analogues and 2ME that indicate a molecular crosstalk between apoptosis and autophagy [36]. In addition, the exact effect that 3MA may have on the cells in the combination treatment would need to be determined (such as induction of p70S6K activity [46]). As inhibitors of PI3K, the loss of this signalling cascade may have further reaching consequences than just autophagy inhibition itself. Further investigation needs to be done to determine whether the increase in autophagosomes observed during ESE-15-ol exposure is due to an increase in autophagy induction or due to abrogated flux (fusion or degradation).

## Conclusions

Data from this study supports the concept that the novel 17-β-estradiol analogue, ESE-15-ol, induces both apoptosis and autophagy in MCF-7 and MDA-MB-231 cells. Results obtained from the morphological studies indicated that 3MA incompletely blocked autophagy, and thereby increased cell survival. Flow cytometry was used to quantify the autophagy inhibition and increased cell viability. This in vitro study revealed that 3MA-mediated inhibition of autophagy increased cell viability of MCF-7 and MDA-MB-231 cells when exposed to ESE-15-ol. Thus it is proposed that the ESE-15-ol-induced autophagic process in MCF-7 and MDA-MB-231 cells contributes to the novel compounds’ cytotoxic effect by inducing programmed cell death type II, as opposed to conferring resistance to the neoplastic cells to drug exposure as a mechanism to compensate for cellular distress. Further quantification of the contribution of autophagy in cell death induction in response to the novel compound, as well as its role in possible tumor resistance will be carried out in future in vitro and in vivo studies.

## Abbreviations

2-ME: 2-methoxyestradiol
2MEOE2bisMATE: 2-methoxyestradiol-bis-sulfamate
3MA: 3-methyladenine
Akt: protein kinase B
ANOVA: analysis of variance
Bcl-2: B cell lymphoma 2
CAII: carbonic anhydrase II
CO: colorado
CO2: carbon dioxide
DMEM: dulbecco’s Minimum Essential Medium Eagle
DMSO: dimethyl sulfoxide
DNA: deoxyribonucleic acid
DR5: death receptor 5
E_2_: estradiol
ESE-15-ol: 2-ethyl-3-*O*-sulpamoyl-estra-1,3,5(10),15-tetraen-17-ol
ESE-15-one: 2-ethyl-3-*O*-sulpamoyl-estra-1,3,5(10),15-tetraen-17-one
ESE-16: 2-ethyl-3-*O*-sulphamoyl-etsra-1,3,5 (10) 16-tetraene
FCS: fetal calf serum
FL1: flow channel 1
FL3: flow channel 3
HepG2: human heptoma cells
HIF-1α: hypoxia-inducible factor -1α
HT1080: fibrosarcoma cell line
IG_50_: half maximum growth inhibitory concentrations
LC3: microtubule-associated protein 1A/1B-light chain 3
MA: Massachusetts
MCF-7: human adenocarcinoma breast cancer cell line
MDA-MB-231: metastatic human adenocarcinoma breast cancer cell line
MDC: monodansylcadaverine
MTOC: microtubule-organising centre
mTor: mechanistic target of rapamycin
PI: propidium iodide
PI3 K: phosphatidylinositide-3-kinase
PI3P: phosphatidylinositol-3-phosphate
PlasDIC: polarization-optical transmitted light differential interference light microscopy
PS: phosphatidylserine
SAC: spindle assembly checkpoint
USA: United States of America
